# Laparoscopic stentless pyeloplasty: An early experience

**DOI:** 10.4103/0970-1591.60444

**Published:** 2010

**Authors:** Vikas Kumar, Anil Mandhani

**Affiliations:** Department of Urology and Renal Transplantation, SGPGIMS, Lucknow, UP, India

**Keywords:** Laparoscopic, pyeloplasty, stent

## Abstract

**Introduction:**

Double J stent has been an important adjunct to laparoscopic pyeloplasty. It is also associated with symptoms and significant morbidity. This study analyses the outcome of transperitoneal laparoscopic pyeloplasty without a double J stent.

**Materials and Methods:**

Sixteen patients of ureteropelvic junction obstruction (age range: 1.5-50 yrs) were selected to undergo transperitoneal stentless laparoscopic pyeloplasty after obtaining the informed consent from August 2004 to December 2008. Ten patients were under the age of 14 years (pediatric age group). Some additional steps in the standard technique of laparoscopic pyeloplasty included anatomical spatulation of the ureter to avoid rotation, temporary splinting while suturing ureteropelvic junction and ensuring water tightness of suture line. Preoperative differential renal function, operative time, post operative complications (pain, drain output, fever), hospital stay and renal functional outcomes (Tc^99^ DTPA) were recorded.

**Results:**

The median age of the pediatric age group was eight (1.5-14) years and adult group-27 (20-50) years. Median operative time was 100 min (72-140) in pediatric and 110 min (90-138) in adult group. The preoperative ipsilateral differential renal function ranged from 16-45% and 16-50% in pediatric and adult groups respectively. Five of the 10 pediatric patients had persistent leak of urine for which stenting was done in four and ureteric re-implantation in one. Only one of the six adult patients (who had secondary UPJO following Endopyelotomy) needed postoperative stenting for persistent urinary leak.

**Conclusions:**

Though the need for postoperative stenting is high in smaller children, stentless laparoscopic pyeloplasty can be considered in adult patients with primary ureteropelvic junction obstruction.

## INTRODUCTION

Although “Anderson-Hynes” dismembered pyeloplasty was first described for the treatment of an obstructed retrocaval ureter in 1949,[1] it is currently the gold standard surgery for ureteropelvic junction obstruction (UPJO), with a success rate greater than 90%.[2] The treatment of UPJO has changed considerably in recent years with the development of laparoscopy and endopyelotomy. Schuessler et al.,[3] first performed a laparoscopic pyeloplasty in 1993 and since then it has been established as a valid technique to correct UPJO.

An important adjunct to laparoscopic pyeloplasty is the placement of a double J stent across the ureteropelvic junction (UPJ) either retrograde[4] or antegrade.[5] Double J stent, however, acts as a splint across the anastomosis and is not free from symptoms. Many patients suffer from severe stent-related symptoms necessitating their early removal. In a study, more than 80% of patients experienced stent-related pain affecting daily activities, 32% experienced sexual dysfunction, and 58%, reduced work capacity and negative economic impact.[6] A complication rate of 94% was reported in another study.[7]

Evidence based literature supports the practice of stentless open pyeloplasty in uncomplicated cases.[8] However, in laparoscopic pyeloplasty, it is still a traditional practice to stent the anastomosis.

As part of our continuing efforts to further address these issues, without compromising on surgical outcomes, we share our initial experience of avoiding stent in transperitoneal laparoscopic pyeloplasty, and thus, better justify the objective of laparoscopic procedures.

## MATERIALS AND METHODS

From August 2004 to December 2008, we randomly selected 16 patients of UPJO (age range 1.5-50 yrs), after explaining the procedure and the need for stent placement, to undergo stentless transperitoneal laparoscopic pyeloplasty (Anderson-Hynes technique) at our institution. Ten patients were under the age of 14 years (pediatric age group). All patients were preoperatively assessed with history, physical examination, abdominal ultrasound, Intravenous urogram (IVU), and diuretic renal scan. The decision for not using stent was strictly based on surgeon's preference and not on grade of hydronephrosis, age of patient, differential renal function, previous abdominal surgery or build of the patient.

**Figure 1:**
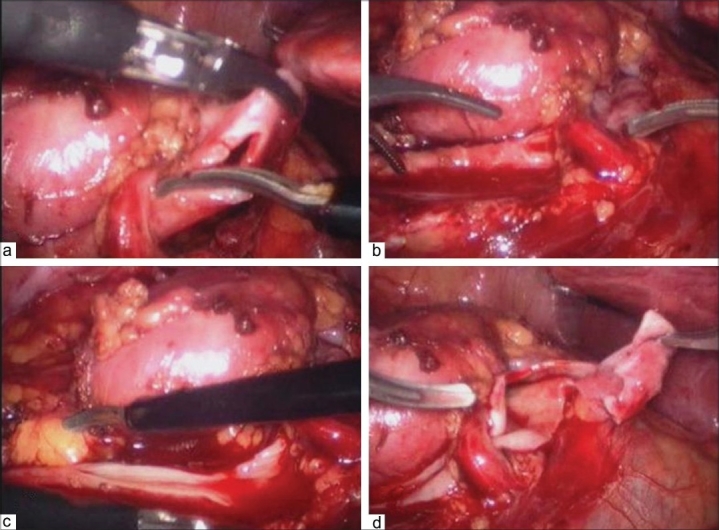
(a-d) Anatomical spatulation of the ureter in the presence of a crossing vessel

Laparoscopic pyeloplasty was done using three ports in 10 patients and four ports in six patients. All surgeries were performed by a single surgeon. Anastomosis was done with 4/0 vicryl in 10 pts, 5/0 vicryl in four and 3/0 vicryl in two patients. All cases had dismembered pyeloplasty. To avoid rotation of ureter, anatomical spatulation technique was used as previously described.[9] Anatomical spatulation also avoids handling of the ureter for making an incision once it is dismembered. It could be done in presence of a crossing vessel too [Figure 1]. Briefly, in this technique, after giving a nick in the renal pelvis just proximal to the UPJ, scissors are directed towards the ureter, which is then spatulated on the lateral side towards the kidney. Ureter is then dismembered.

### Technique of Stentless Ureteropelvic Junction Anastomosis:

**Figure 2:**
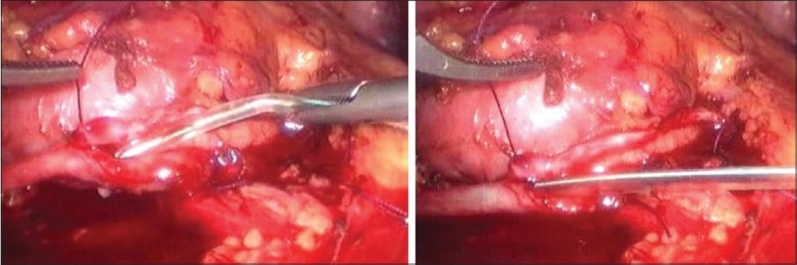
(a-b) Feeding tube being used as a temporary splint across the ureteropelvic junction

**Figure 3:**
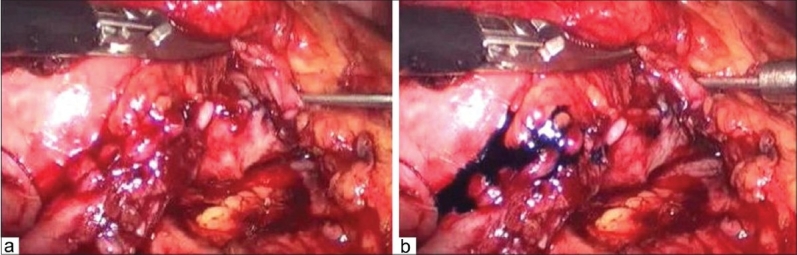
(a) Testing the water tightness of the suture line with saline mixed methylene blue; (b) Reinforcing the suture line at the site of leak

After completion of posterior suture line at ureteropelvic junction, an infant feeding tube (5-6 fr in pediatric group and 7-8 fr in adults) was placed in the ureter as a temporary splint and as soon as the anterior suture line of ureteropelvic junction was completed, this splint was taken out [Figure 2]. After this, rest of the renal pelvis was sutured. Water tightness of the suture line was checked by injecting saline mixed with methylene blue dye into the renal pelvis to distend it with a laparoscopic needle [Figure 3a]. Any leak was further reinforced with a separate suture[Figure 3b]. A tube drain was placed. The drain was removed in the post operative period when its 24 hour output decreased to <25-50 ml.

Age, gender, preoperative differential renal function, operative time, operative findings, post operative complications (pain, drain output, fever), hospital stay and any improvement in the renal functional outcomes (Tc^99^ DTPA) were recorded. Operative time was measured from insertion of first port to closure of last port.

The decision for postoperative double J stenting was made if there was persistent high drain output for more than five days. The follow-up schedule included a visit at two weeks with urine analysis and clinical examination. Diuretic renogram was done at three months of surgery and later every 6-12 months.

## RESULTS

Of the 16 patients (age range 18 months - 50 years), 15 had primary UPJO and one had secondary UPJO (following Endopyelotomy). Ten patients were in the pediatric age group with a median age of eight yrs (range 1.5-14 yrs) and male to female ratio of 9:1. Six adult patients had a median age of 27 yrs (range 20-50 yrs) with male to female ratio of 2:1.

Nine patients had left and seven had right sided UPJO. All patients were symptomatic and had proven significant obstruction on Tc^99^-DTPA renal scan. None of the pediatric patients had history of antenatal diagnosis.

Median operative time was 100 min (72-140 min) in children and 110 min (90-138 minutes) in adults. Three patients had intrarenal pelvis with one having malrotated kidney. Crossing vessel was present in six patients. Transposition was done in all. None of the patients had conversion to open surgery. Urethral catheter was removed on the second day of surgery. Drain output varied from nil to 1350 ml on first postoperative day that decreased gradually in most patients and drain was removed from third to fifth postoperative day, when output decreased to less than 25-50 ml. Median time to bowel movement was 36 hrs (24-40 hrs). None of the patients had any signs of peritonitis in the postoperative period. In one of the pediatric patients, drain slipped out accidentally, but there was no evidence of collection on ultrasound.

**Figure 4:**
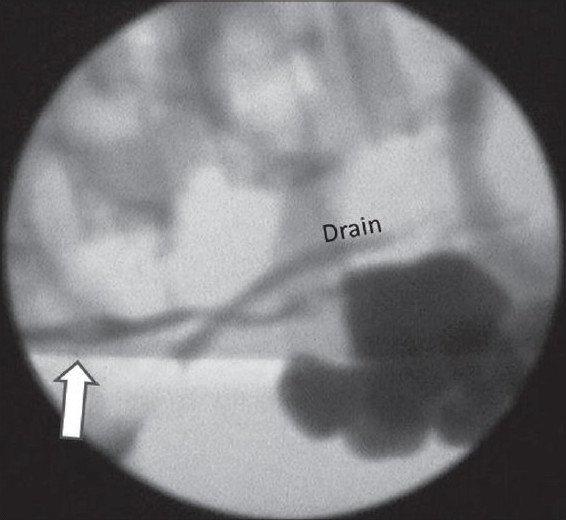
Clot in the ureter (marked with white arrow) causing obstruction and urinary leak (visible through the drain)

The median hospital stay (calculated as time after the surgery) was 7.5 days (3-16 days). One out of six (16.6%) adult patients (who had secondary UPJO following endopyelotomy) needed postoperative stenting due to persistent urinary leak. Five of the 10 pediatric patients (50%) had persistent leak for which stenting was done in four and ureteric re-implantation in one. The reason for re-implantation was presence of a concomitant ureterovesical junction obstruction which was overlooked as retrograde pyelography was not done routinely in all the patients undergoing laparoscopic pyeloplasty. Of the four pediatric patients who required stenting, two children in initial experience had hematuria and clot colic which was responsible for persistent drainage [Figure 4]. Two children had very large pelvis, which, possibly due to improper reduction of the pelvis with relatively low function (26 and 29% differential renal function), might not have created good bolus of urine to flow across the UPJ. Two of these four pediatric patients were under the age of two years.

There was no relation of postoperative need for stenting and crossing vessels. The median preoperative differential renal function of the involved kidney was 31% (16-45%) in children and 39% (16-50%) in adults. All patients had relief of symptoms and 15 (93.7%) had shown nonobstructed drainage with improved differential renal function on post operative renal scan at the median follow up of 40 months (6-58 months) [Table 1]. One patient who showed equivocal obstruction on the postoperative renal scan was also relieved of symptoms.

**Table 1:** Demographic profile and the outcome of 16 patients of stent less pyeloplasty

Parameter	Pediatric patients (%)	Adult patients (%)
Number of patients	10	6
Median age in years (range)	8 (1.5-14)	27(20-50)
Median (range) operative time (min)	100(72-140)	110(90-138)
Patients needing post operative intervention	5(50)	1(16.6)
Median pre-operative renal function of the ipsilateral unit	31(16-45)	39(16-50)
Improvement in post operative renal scan(no. of patients)	9(90)	6(100)
Symptomatic relief	10(100)	6(100)

## DISCUSSION

Ureteropelvic junction obstruction was traditionally managed by open pyeloplasty via a retroperitoneal approach. With the advent of minimally invasive surgery (MIS), there is an increasing role for the laparoscopic approach in performing this operation. With its ability to replicate each step of open surgical procedure, laparoscopic approach provides a combination of equivalent success rates of open surgery (>90%) and advantages of decreased pain, improved cosmesis, shorter hospital stay and an early return to full activity.

Laparoscopic pyeloplasty is continuously evolving with various modifications to simplify the technique to make it a truly minimally invasive approach. There has been an ongoing debate on the merits of intubated versus non-intubated (stent less) repair of UPJO done either by laparoscopic or open technique. The advantages of stent placement following pyeloplasty include lowering the risk of urinoma formation, ensuring urinary drainage, maintaining ureteric calibre and anastomotic alignment, and lowering the impact of postoperative edema at the anastomotic site.[8] More recently, there seems to have been a trend towards non-stented repairs.[10,11]

It is of interest to mention the comment from Anderson and Hynes on their technique- “We are convinced that the so called splinting of any anastomosis is not only unnecessary but it is against all the principles of plastic procedure, as it leads to infection and fibrosis at the line of suture and subsequent stricture. The line of anastomosis should be wide enough and so fashioned as to render any subsequent contraction innocuous”- they did not drain the renal pelvis or use a transanastomotic tube/stent.[1]

This original description of non-stented dismembered pyeloplasty was followed by several reports debating the need of anastomotic stenting but unfortunately the literature remains inconclusive on this issue.

Stent has been found to be associated with stent syndrome (defined as dysuria, frequency, flank pain and hematuria commonly seen with short term placement of ureteral stents), interfere with daily activities and result in reduced quality of life. In their study on indwelling ureteral stents, Joshi *et al*., reported that 78% patients had bothersome urinary symptoms that included storage symptoms, incontinence and hematuria.[6] More than 80% of patients experienced stent related pain affecting daily activities, 32% reported sexual dysfunction, and 58% reported reduced work capacity and negative economic impact. The mean Euro quality of life (EuroQoL) utility values, which indicate patient satisfaction with treatment, were significantly reduced following stent insertion.[6] Other potential problems include migration, encrustation, retained or forgotten fragments, exposure of the upper tract to high pressure during micturition, flank pain and increased urinary infections.[7,12,13]

Though stent can be removed under local anesthesia in adults, its removal requires general anesthesia in children. Various attempts have been made to avoid a repeated exposure to general anesthesia for stent removal in pediatric population. Mykulak *et al*.,[14] and Macaluso *et al*.,[15] described the use of magnetic tip double J catheter but this approach had fallen out of favor with time due to technical difficulties with removal. Taveres *et al*., presented a technique for inserting an internal-external nephroureteral antegrade stent during laparoscopic pyeloplasty which can be easily removed in clinic as outpatient, but authors warned about profuse bleeding after puncture of parenchyma, and other risks of any percutaneous renal access including injury to nonvisualized wall of large bowel.[16]

In the present series, only one out of six adult patients needed postoperative stenting for persistent increased drain output; this was the only patient in the series with history of previous surgery (endopyelotomy). None of the other five patients experienced any postoperative complications and/or required stenting. Thus we achieved 100% success in adults with primary UPJO in terms of surgical outcome and avoiding the stent related problems.

In our pediatric group, four patients needed stenting in the postoperative period and one needed ureteric reimplantation and the psoas hitch procedure. Two of these patients needing intervention in the postoperative period were under the age of two years, thus, questioning the feasibility of stent less surgery in very young children. This issue was also addressed by Woo *et al*., in their study on the impact of internal stenting on the surgical outcome of dismembered pyeloplasty in infants under the age of 12 months. They observed statistically significant increase in urinary leaks, length of hospital stay and need for repeat pyeloplasty in non stented versus the stented group and concluded that use of internal ureteral stenting in such cases led to a dramatic reduction in operative morbidity; however the mode of surgery in this study was open pyeloplasty.[17]

As laparoscopic pyeloplasty is technically challenging in smaller children, with a dedicated pediatric urologist and technical refinement, it might be possible to reduce the chance of re-stenting.

We attribute our success results in patients with primary UPJO to meticulous watertight suturing and anatomical spatulation preventing rotation of the ureter. Minor rotation of the ureter would not mean much, if there is a double J stent *in situ*, but this would implicate the result if there is no stent. Similarly, good hemostasis is mandatory to avoid hematuria and formation of clots, which can hinder the drainage of urine. Temporary splinting across the ureteropelvic junction eliminates the chance of taking opposite wall suture, compromising the patency of the UPJ.

## CONCLUSIONS

Stentless pyeloplasty is a feasible option and proper spatulation of the ureter, better hemostasis, watertight anastomosis on a temporary splint and better reduction of the renal pelvis would add to the success of the procedure. Performing stent less pyeloplasty in smaller children is technically challenging and the need of postoperative stenting, which requires an additional anesthesia, is high. Stentless pyeloplasty in adult patients with primary ureteropelvic junction obstruction looks promising and requires to be validated with larger number of patients.
